# Neurology pioneers in Japan

**DOI:** 10.1055/s-0044-1791661

**Published:** 2025-01-15

**Authors:** Hélio A. Ghizoni Teive, Léo Coutinho, Francisco E. C. Cardoso, Shoji Tsuji

**Affiliations:** 1 Universidade Federal do Paraná, Complexo do Hospital de Clínicas, Departamento de Medicina Interna, Serviço de Neurologia, Unidade de Distúrbios de Movimento, Curitiba PR, Brazil.; 2 Universidade Federal do Paraná, Complexo do Hospital de Clínicas, Departamento de Clínica Médica, Programa de Pós-Graduação em Medicina Interna e Ciências da Saúde, Grupo de Doenças Neurológicas, Curitiba PR, Brazil.; 3 Universidade Federal de Minas Gerais, Hospital de Clínicas, Departamento de Medicina Interna, Serviço de Neurologia, Unidade de Distúrbios de Movimento, Belo Horizonte MG, Brazil.; 4 The University of Tokyo Hospital, Department of Neurology, Tokyo, Japan.; 5 International University of Health and Welfare, Institute of Medical Genomics, Otawara, Japan.

**Keywords:** History of Medicine, History, 19 ^th^ Century, Neurology, Neurosciences, Japan, História da Medicina, História do Século XIX, Neurologia, Neurociências, Japão

## Abstract

The pioneers of neurology in Japan were professors Hiroshi Kawahara and Kinnosuke Miura. Kawahara published the first description of progressive bulbar palsy and wrote the first neurology textbook in Japan. Miura, on the other hand, published studies about amyotrophic lateral sclerosis, in addition to participating in the founding of the Japanese Society of Neurology. The influence of European neurology, particularly French and German, in the figures of Professor Jean-Martin Charcot and Professor Erwin Bälz, was fundamental in the consolidation of neurology in Japan.

## Resumo

Os pioneiros da Neurologia no Japão foram os professores Hiroshi Kawahara e Kinnosuke Miura. Kawahara publicou a primeira descrição de paralisia bulbar progressiva e escreveu o primeiro livro-texto de Neurologia no Japão. Já Miura publicou estudos sobre esclerose lateral amiotrófica, além de participar da fundação da Sociedade Japonesa de Neurologia. A influência da Neurologia europeia, particularmente francesa e alemã, nas figuras dos Professores Jean-Martin Charcot e Erwin Bälz foi fundamental na consolidação da Neurologia no Japão.

## INTRODUCTION

The dawn of medicine in Japan is linked to the introduction of Chinese medicine and Buddhism, in 573 AD. 1 Japan long remained closed to contact with foreigners. In 1543, contact with Western culture occurred, initially with the Portuguese and later with the Dutch, particularly with the presence, among others, of the naval surgeon Johannes Lydius Catherinus Pompe van Meerdervoort (1829–1908). 1 2 3 4

Starting in the Meiji era, in 1867, Western medicine had a greater influence on Japanese medicine, especially Germany, France, and the United Kingdom. 1 2 3 4 5 The new government fostered a fruitful cooperation with Imperial Germany, with Japanese students going to Germany and German professors going to Japan. Erwin Bälz, a disciple of Professor Carl Reinhold August Wunderlich (1815–1877) of the University of Leipzig, went to Japan as part of this partnership and served as Professor of Medicine at the Tokyo Medical School, now the University of Tokyo, for 25 years. 1 2 3 4 5 Bälz's teachings sparked the interest of two Japanese medical students, Hiroshi Kawahara and Kinnosuke Miura, in the specialty of neurology. 6 7 8 9 10 11 12

This review highlights the contributions of Professors Kawahara and Miura, two of the main neurological pioneers in Japan, to the development of Japanese neurology.

## PROFESSOR HIROSHI KAWAHARA (1858–1918)

**Figure 1:**
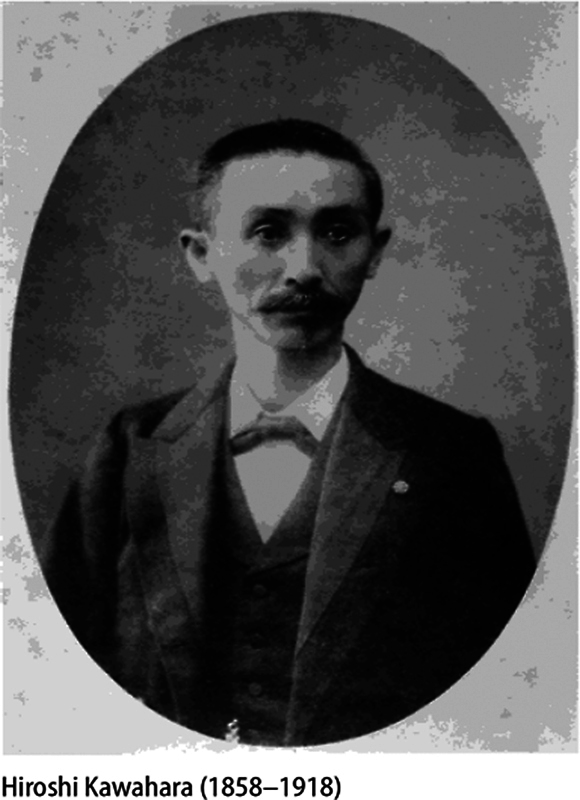
Hiroshi Kawahara (1858–1918). Licensed under a public domain mark.

Professor Hiroshi Kawahara ( Figure 1 ) was born in Omura, Kyushu, in 1858. He began his medical studies at the medical school in Nagasaki, founded under the guidance of professor Pompe van Meerdervoort, in 1871. 6 9 10 11 12

Later, Kawahara transferred to the medical school in Tokyo in 1874, which is now the Faculty of Medicine of the University of Tokyo, where he was influenced by the teachings of Professor Erwin Bälz. He graduated in 1883 despite having tuberculosis. 6 9 10 11 12

Kawahara's interest was initially in internal medicine, but he later became enthusiastic about neurology. Although Kawahara did not have a chance to work abroad, he extensively read several books and literature published in France, England, and Germany, indicating that he had rich information on Western Neurology. 6 9 10 11 12

After his graduation and training under Professor Bälz, he transferred to Saga Medical School, in Kyushu, in July 1883, and then to the Aichi Medical School, now the Nagoya University School of Medicine, in October 1883. He was initially a professor of pathological anatomy and then of internal medicine, until 1897, when he retired due to pulmonary tuberculosis, which would lead to his death in 1918, at the age of 59. 6 9 10 11 12

Kawahara is the author of the first textbook of neurology in Japan, published in 1897, 13 and in this book, he gave a brilliant description, though not the first one, historically, of amyotrophic lateral sclerosis (ALS) in Japanese patients.

His greatest academic contribution was the pioneering description of a family illness called “progressive bulbar palsy”, in 1897. 14 The disease is today defined as X-linked recessive bulbar-spinal muscular atrophy, known as Kennedy disease, or more recently Kennedy-Alter-Sung syndrome. 9 10 11 12 15 16 17 18

These authors published a study in 1968, analyzing two families, with 11 men, and defined the disease as progressive proximal spinal and bulbar muscular atrophy of late onset. 16 17 18 However, Kawahara described the same disease 71 years earlier, reporting 3 patients from the same family (2 brothers and a maternal uncle), with clear X-linked recessive inheritance. 14 15

## PROFESSOR KINNOSUKE MIURA (1864–1950)

**Figure 2:**
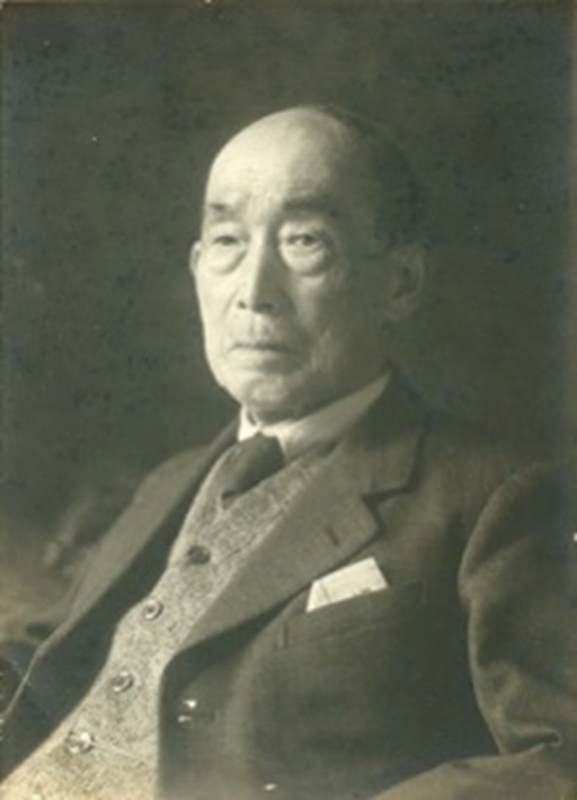
Kinnosuke Miura (1864–1950). Courtesy of Professor Yasusada Miura, grandson of Prof. Kinnosuke Miura.

Kinnosuke Miura graduated from the University of Tokyo in 1887 ( Figure 2 ). 7 8 9 11 12 13 After 2 years of training in internal medicine with Professor Erwin Bälz, Miura began his activities as a private physician to Prince Takahito Arisugawa, of the Japanese imperial family. 7 8 9

Miura, aged 25 in 1889, accompanied members of the Japanese royal family on their European tour for 2 years (1889–1890). He visited various Neurology services, initially in Berlin, Marburg, and Heidelberg, in Germany. Then in Paris, he did an internship at the neurology service of Professor Jean-Martin Charcot (1825–1893). 7 8 9 After the return of members of the imperial family to Japan, Miura remained in Paris for 2 more years (1891–1892) as an intern at Professor Charcot's clinic. 7 8 9

Miura had great admiration and deep affection for Charcot, and often repeated that “it was Charcot who examined neurology patients as a real neurologist.” 8 In an article published in 1993, Professor Miura's son, Y. Miura, recalls his father's admiration for Master Charcot, emphasizing the anatomo-clinical method, rigorous clinical observation, and meticulous study of neurological signs. 8 After the death of Jean-Martin Charcot, Professor Miura continued a good relationship with Charcot's disciples, especially Pierre Marie. 7 8 9

Professor Charcot's great influence on Professor Miura's career can be gauged by Miura's major contribution to Japanese Neurology. 7 8 9 He described cases of amyotrophic lateral sclerosis, Marie ataxia, and cases of a disease called kubisagari (“head-dropping”). 7 8 9

In 1902, Miura, together with his colleague Shuzo Kure, founded the Japanese Society of Neurology. The journal of the society, the first journal of Neurology in Japan, called *Neurologia Japonica* , was published in the same year. 7 8 9 12 Still in 1902, Miura presented his description of ALS in the journal, 19 whose publication widely established the entity of ALS in Japan. 18

He died at the age of 87, in 1950, suddenly, while heading to a patient's house and was found unconscious in a street. The necropsy examination revealed the presence of a pontine intracerebral hemorrhage. 7 8 9

## AFTER KAWAHARA AND MIURA

Kawahara and Miura's advances were carried along by the next generation of neurologists. The works of Kawahara on X-linked recessive bulbar-spinal muscular atrophy were further added by contributions of Mukai and Hirayama. 6 Miura, on the other hand, as professor at the University of Tokyo, trained leading professors in the field, such as Seizō Katsunuma, Ken Kure, and Shigeo Okinaka. 1

In conclusion, Professors Hiroshi Kawahara and Kinnosuke Miura were two of the main pioneers of Japanese Neurology. Both were trained in internal medicine by German Professor Erwin Bälz at the University of Tokyo. Charcot's and Bälz's influence on Japanese Neurology was undeniable, considering the importance of Miura to the development of Neurology in Japan, by co-founding the Japanese Society of Neurology and the first publication of a Neurology journal in Japan. We highlight the importance of new historical data, such as Kawahara's contributions to spinal bulbar atrophy, to the advancement of scientific knowledge.
